# Increasing UCP2 expression and decreasing NOX1/4 expression maintain chondrocyte phenotype by reducing reactive oxygen species production

**DOI:** 10.18632/oncotarget.18908

**Published:** 2017-07-01

**Authors:** Yansong Miao, Yuefu Dong, Ping Huang, Xiang Zhao, Zhenyu Huang, Jufang Yao, He Li, Qingrong Xu

**Affiliations:** ^1^ Department of Orthopaedics, Ren Ji Hospital, School of Medicine, Shanghai Jiao Tong University, Shanghai, China; ^2^ Department of Joint Surgery, The First People's Hospital of Lianyungang, Lianyungang, China; ^3^ Department of Cerebral Surgery, Tong Ren Hospital, School of Medicine, Shanghai Jiao Tong University, Shanghai, China; ^4^ Department of Animal Facility, Ren Ji Hospital, School of Medicine, Shanghai Jiao Tong University, Shanghai, China; ^5^ Department of Traditional Chinese Medicine, Ren Ji Hospital, School of Medicine, Shanghai Jiao Tong University, Shanghai, China

**Keywords:** UCP2, NOX1/4, PGC-1α, TFAM, chondrocyte phenotype

## Abstract

The aim of this study is to demonstrate that improving the mitochondrial function can inhibite the loss of chondrocyte phenotype by regulating the expression of uncoupling protein 2(UCP2) and NADPH oxidase1/4(NOX1/4) to reduce the production of reactive oxygen species(ROS). The effects of mitochondrial biogenesis “master regular” peroxisome proliferator-activated receptor gamma coactivator-1α (PGC-1α), mitochondrial transcriptional factor A (TFAM), UCP2, and NOX1/4 on chondrocyte phenotype was examined. It was found that when the chondrocyte phenotype was lost, PGC-1α, UCP2, and TFAM expression decreased, while NOX1/4 expression increased. Inhibiting UCP2 expression promoted the loss of chondrocyte phenotype, and inhibiting NOX1/4 relieved the loss of the chondrocyte phenotype. After activating the PGC-1α-TFAM pathway, UCP2 increased and NOX1/4 decreased, which suppressed loss of the chondrocyte phenotype. After inhibiting NOX1/4, UCP2 expression increased. Increasing and decreasing UCP2 and NOX1/4 expression, respectively, helps maintain the chondrocyte phenotype and improve mitochondrial functioning by reducing reactive oxygen species production.

## INTRODUCTION

Loss of chondrocyte phenotype is followed by loss of normal articular cartilage function [1–2]. Elevated col1, decreased expression of col2, aggrecan, and Sox9, and changes in fibroblast morphology lead to poor fibrocartilage formation, which results in losing the original chondrocyte phenotype [3–7]. Increased oxidative stress caused by excessive production of reactive oxygen species (ROS) has also been shown to promote activation of the inflammatory machinery, which in turn promotes chondrocyte apoptosis in osteoarthritis (OA) [8]. Oxidative stress leads to the onset of aging or reduces survival due to increasing the production of ROS [9, 10]. Similarly, exposure to ionizing radiation and subsequent production of ROS induces chondrocyte senescence [11].

Expression of PGC-1α is reduced in aging mouse knee cartilage [12, 13]. PGC-1α suppresses cellular oxidative stress by up-regulating the ROS depletion system (antioxidase) in mitochondria, including manganese superoxide dismutase (MnSOD or SOD2) in vascular endothelial development and homeostasis [14, 15]. PGC-1α can upregulate ATP levels [16], as well as improve mitochondrial function by promoting transcription factor A, mitochondrial (TFAM). It is not known if the PGC-1α-TFAM pathway can inhibit the loss of chondrocyte phenotype.

The putative signaling function of UCP2 is thought to stem from its capacity to control mitochondrial ROS emission [17, 18]. Superoxide anion radicals are produced by the complex enzyme NADPH, which catalyzes the formation of superoxide anion and NAPDH oxidase, which is a membrane-bound enzyme [19]. NADPH oxidase exists both in and outside the mitochondria. NOX1 is mainly stored in the cytoplasm, while NOX4 is stored in both the cytoplasm and mitochondria. As NADPH oxidase increases the production of ROS, it leads to imbalance in mitochondrial uncoupling (mainly the role of UCP2), which is the main cause of hypoxia in reduction of renal mass (RRM) [20]. Interactions between UCP2 and NOX in chondrocytes have not been reported.

## RESULTS

### When chondrocyte phenotype was lost, PGC-1α, UCP2, and TFAM expression decreased, NOX1/4 expression increased, and ROS production increased

**Figure 1:**
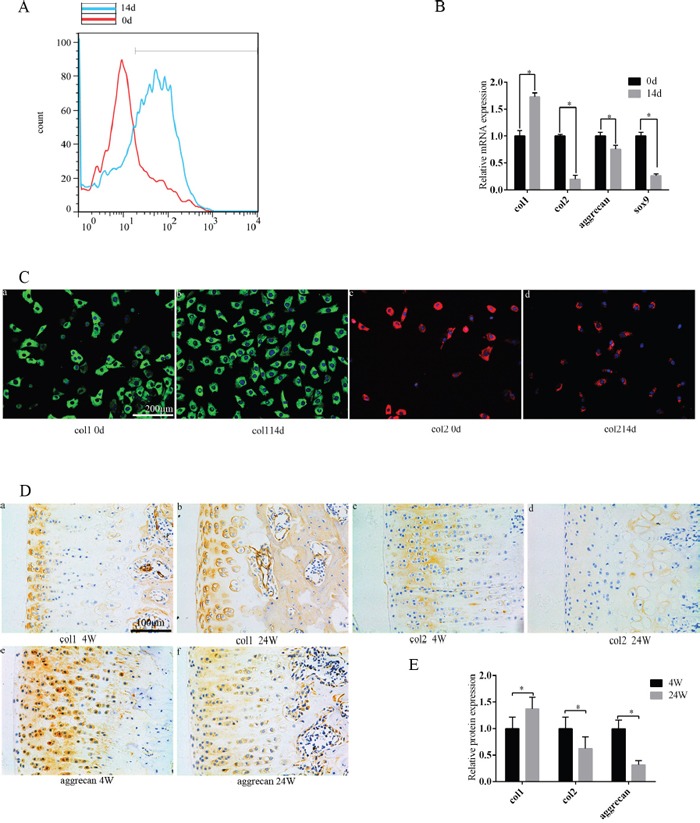
During the culture of chondrocytes *in vitro*, the chondrocyte phenotype was lost and the production of ROS was increased As the age of the rats progressed, the chondrocyte phenotype was also lost in the articular chondrocytes. **(A)** Compared with primary cells, ROS production increased in chondrocytes after 14 days. **(B)** Compared with primary cells, the chondrocyte phenotype was lost after 14 days according to PCR technique. **(C)** Compared with primary cells, the expression of col1 and col2 was altered after 14 days according to the immunofluorescence technique. **(a/b)** Col1 expression. **(c/d)** Col2 expression; bar = 200 μm. **(D)** Compared to 4 week old rats, the chondrocyte phenotype in the 24 week old rat was lost according to immunohistochemistry. **(a/b)** Col1 expression. **(c/d)** Col2 expression. **(e/f)** Aggrecan expression;bar = 100 μm. **(E)** Immunohistochemistry results are presented as mean ± SD, and are representative of three independent experiments.

**Figure 2:**
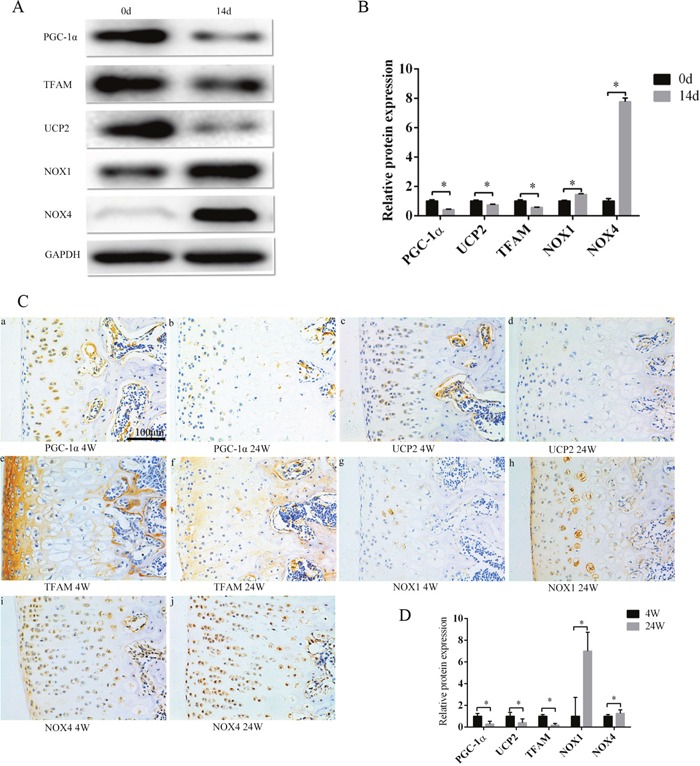
PGC-1α, TFAM, and UCP2 expression decreased, and NOX1/4 increased during the culture of chondrocytes *in vitro*, and as the rats aged **(A)** Compared with primary cells, the expression of PGC-1α, TFAM, and UCP2 was decreased, and NOX1/4 increased after a 14-day culture, according to Western blot. **(B)** Western blot results are mean ± SD, and are representative of three independent experiments. **(C)** The expression of PGC-1α, TFAM, and UCP2 was decreased, and NOX 1/4 increased in the chondrocytes of 24 week rats compared with 4 week old rats. Bar = 100 μm. **(D)** Immunohistochemistry results are the mean ± SD, and are representative of three independent experiments.

Compared with primary chondrocytes, ROS production increased in the 14-day culture (Figure 1A). Isolated chondrocytes may begin to lose their phenotype in conventional subcultures, which leads to severe impairment of their intrinsic properties [4]. We found that the chondrocytes that cultured *in vitro* for 14 days have different degrees of chondrocyte phenotype loss compared with the original generation of chondrocytes. As culture time progressed, PCR showed that col2, Sox9, and aggrecan expression decreased, and col1 expression increased (Figure 1B). Col2 and col1 expression also decreased and increased, respectively, at the protein level (Figure 1C). In animal models, the expression changes of col2, col1, and aggrecan were verified again. Col1 was increased, col2 and aggrecan was decreased when comparing 4 week rats with 24 week rats (Figure 1D, 1E). PGC-1α, TFAM, and UCP2 expression decreased in the 14-day culture compared with primary chondrocytes, while NOX1/4 increased (Figure 2A, 2B). Identical trends were observed when comparing 4 week rats with 24 week rats (Figure 2C, 2D).

### The up-regulation of PGC-1α-TFAM pathway can inhibit the loss of chondrocyte phenotype and reduce ROS production

**Figure 3:**
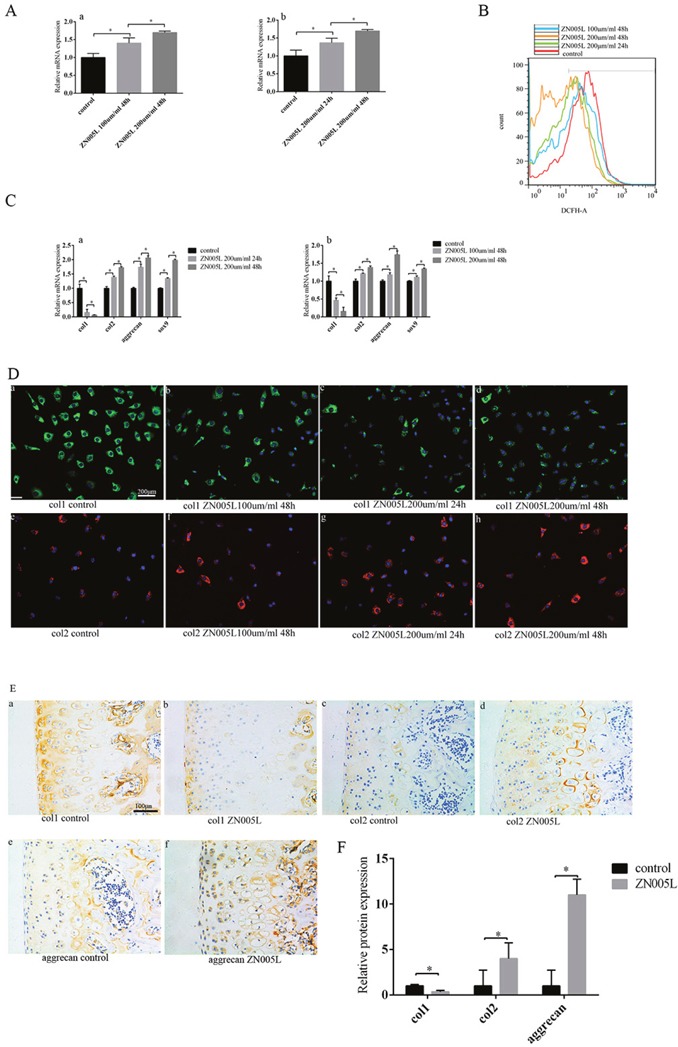
Activating PGC-1α expression can inhibit the loss of chondrocyte phenotype by reducing ROS production *in vitro*, and after injecting ZN005L into the articular cavity **(A)** As ZN005L concentration and functional duration increased, PGC-1α expression increased. **(a)** The relationship between the concentration of ZN005L and the expression of PGC-1α. **(b)** The relationship between ZN005L duration and PGC-1α expression. **(B)** As ZN005L concentration and functional duration increased, ROS production decreased according to cell flow technology. **(C)** As ZN005L concentration and functional duration increased, the loss of chondrocyte phenotype was inhibited. **(a)** The relationship between ZN005L concentration and chondrocyte phenotype. **(b)** The relationship between ZN005L duration and chondrocyte phenotype. **(D)** After activating PGC-1α expression, col2 and col1 expression increased and decreased respectively according to the cell fluorescence technique. **(a/b/d)** The relationship between col1 expression and ZN005L concentration. **(a/c/d)** The relationship between col1 expression and ZN005L duration. **(e/f/h)** The relationship between col2 expression and ZN005L concentration. **(e/g/h)** The relationship between col2 expression and ZN005L duration; bar = 200 μm. **(E)** The loss of chondrocyte phenotype was inhibited after injecting ZN005L into the knee joint of SD rats, as determined by immunohistochemistry. **(a/b)** Col1 expression. **(c/d)** Col2 expression. **(e/f)** Aggrecan expression; bar = 100 μm. **(F)** Immunohistochemistry results are mean ± SD, and are representative of three independent experiments.

ZN005L can increase the expression of PGC-1α mRNA [21]. Chondrocytes cultured *in vitro* were divided into several groups and given different doses of PGC-1α agonist ZN005L. As ZN005L concentration and duration increased, PGC-1α expression increased (Figure 3A). Adding ZN005L also decreased ROS production (Figure 3B) according to FACS. As ZN005L concentration increased and duration extended, col1 expression gradually decreased, while col2, Sox9, and aggrecan expression increased (Figure 3C). When compared to control, chondrocyte phenotype loss was inhibited more with greater concentration and time duration of ZN005L (Figure 3C). At the protein level, col1 and col2 expression decreased and increased, respectively (Figure 3D). Compared to the control group, all the same changes in expression were observed in SD rats injected with ZN005L (Figure 3E, 3F).

**Figure 4:**
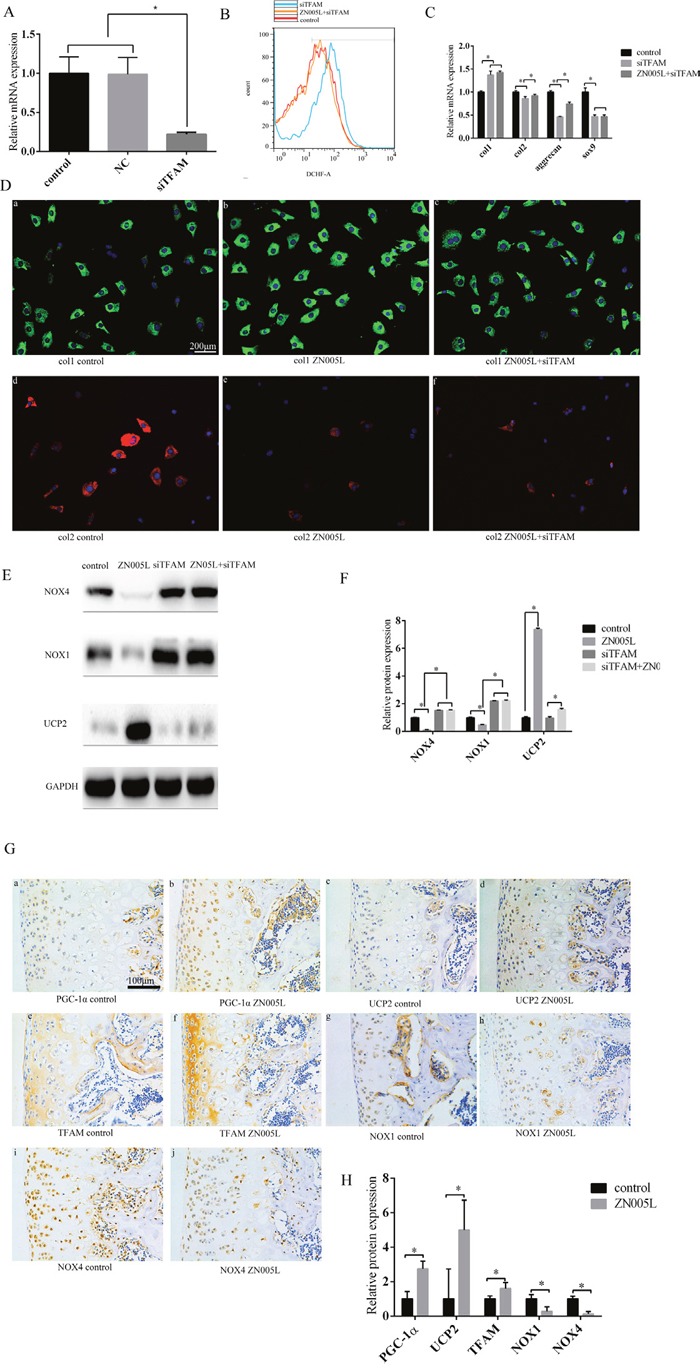
Activating the PGC-1α-TFAM pathway can inhibit the loss of chondrocyte phenotype and decrease ROS production **(A)** After interfering with the expression of TFAM, TFAM expression decreased. **(B)** Decreasing TFAM can increase ROS production; compared with the siTFAM group, ROS production decreased in the siTFAM+ZN005L group. **(C)** After decreasing TFAM expression, the chondrocyte phenotype was lost. Compared with the siTFAM group, col2 and aggrecan expression increased slightly in the ZN005L+siTFAM group according to PCR; compared with the siTFAM group, col1 and SOX9 expression did not change in the ZN005L+siTFAM group according to PCR. **(D)** After decreasing TFAM expression, col1 expression increased and col2 expression decreased. Compared with the siTFAM group, col1 and col2 expression did not change in the siTFAM+ZN005L group according to cell fluorescence technique. **(a/b/c)** Col1 expression. **(d/e/f)** Col2 expression. Bar = 200 μm. **(E)** After decreasing TFAM expression, NOX1/4 expression increased and UCP2 expression did not change. Compared with the siTFAM group, UCP2 expression slightly increased in the siTFAM+ZN005L group according to Western blot. **(F)** Western blot values are mean ± SD, and are representative of three independent experiments. **(G)** After injecting ZN005L into the knee joint cavity of SD rats, TFAM and UCP2 expression increased and NOX1/4 decreased according to immunohistochemistry. **(a/b)** PGC-1α expression. **(c/d)** UCP2 expression. **(e/f)** TFAM expression. **(g/h)** NOX1 expression. **(i/j)** NOX4 expression. Bar = 100 μm. **(H)** Immunohistochemistry values are mean ± SD, and are representative of three independent experiments.

After interfering with TFAM expression by using siRNA, TFAM expression decreased (Figure 4A) and ROS production increased (Figure 4B). At the gene level, col1 expression increased while col2, Sox9, and aggrecan decreased, which was accompanied by the loss of the chondrocyte phenotype. In the siTFAM+ZN005L group, Sox9 and col1 did not change, while col2 and aggrecan expression increased (Figure 4C). At the protein level, the change of col1 was the same as seen in gene expression (Figure 4D). Col2 expression in the siTFAM group did not change when compared with the siTFAM+ZN005L group (Figure 4D). These results indicate that activating the PGC-1α-TFAM pathway can inhibit the loss of chondrocyte phenotype by reducing ROS production.

### PGC-1α-TFAM pathway can increase UCP2 expression and reduce NOX1/4 expression

To understand how PGC-1α-induced ROS reduction inhibits the loss of chondrocyte phenotype, the NOX1/4 and UCP2 levels were observed after adding ZN005L. After adding PGC-1α agonist ZN005L, UCP2 expression increased while NOX1/4 expression decreased compared with the control group. After using siRNA to interfere with TFAM expression, NOX1/4 expression increased and UCP2 displayed no change compared with the control group. However in the ZN005L+ siTFAM group, NOX1/4 expression did not change compared with the siTFAM group, and UCP2 expression increased compared with the ZN005L group (Figure 4E, 4F).

In our animal model, after activating PGC-1α expression by injecting ZN005L, TFAM and UCP2 expression increased, while NOX1/4 expression decreased (Figure 4G, 4H). Activating the PGC-1α-TFAM pathway can reduce ROS production and reverse the loss of chondrocyte phenotype by decreasing and increasing NOX1/4 and UCP2 expression, respectively.

### Inhibiting UCP2 expression promotes the loss of chondrocyte phenotype and increases ROS production

**Figure 5:**
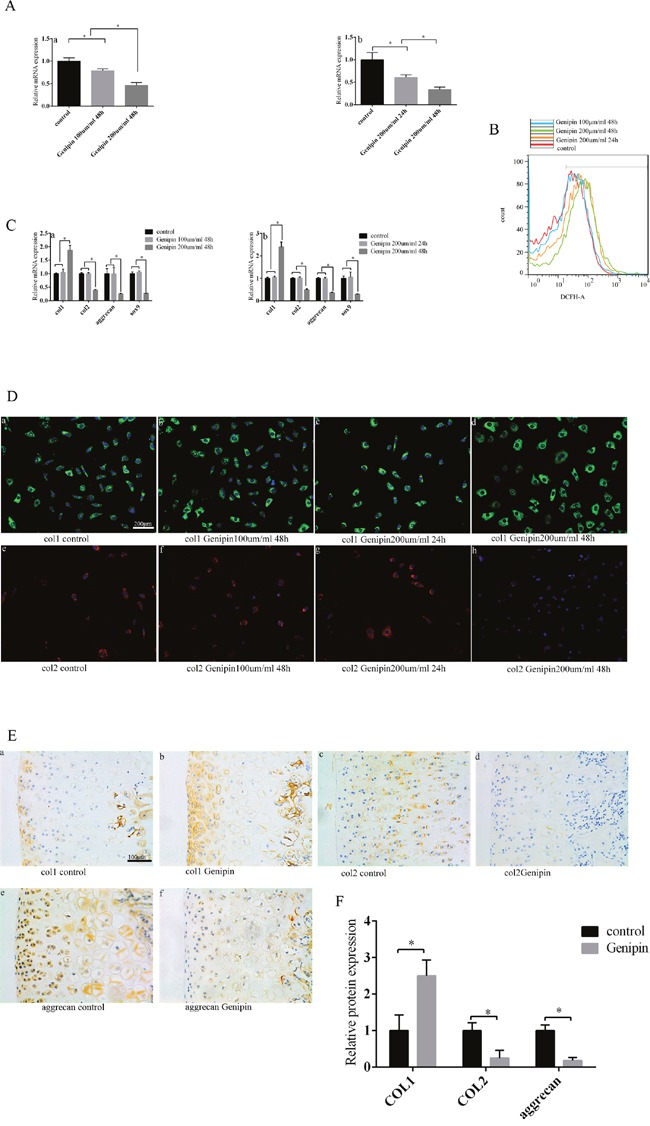
Inhibiting UCP2 expression can increase ROS production and subsequently promote the loss of chondrocyte phenotype **(A)** After increasing genipin concentration and duration, UCP2 expression decreased. **(a)** The relationship between genipin concentration and UCP2 expression. **(b)** The relationship between genipin duration and UCP2 expression. **(B)** After increasing genipin concentration and duration, ROS production increased as determined by cell flow technology. **(C)** After increasing genipin concentration and duration, chondrocyte phenotype was lost. **(a)** The relationship between genipin concentration and chondrocyte phenotype. **(b)** The relationship between genipin duration and chondrocyte phenotype. **(D)** After inhibiting UCP2 expression, col1 expression increased and col2 expression decreased according to the cell fluorescence technique. **(a/b/d)** The relationship between col1 expression and genipin concentration. **(a/c/d)** The relationship between col1 expression and genipin duration. **(e/f/h)** The relationship between col2 expression and genipin concentration. **(e/g/h)** The relationship between col2 expression and genipin duration. Bar = 200 μm. **(E)** The loss of chondrocyte phenotype was promoted after injecting genipin into the knee joint of SD rats as determined by immunohistochemistry. **(a/b)** Col1 expression. **(c/d)** Col2 expression. **(e/f)** Aggrecan expression. Bar = 100 μm. **(F)** Immunohistochemistry results are the mean ± SD, and are representative of three independent experiments.

Genipin acts as a cell permeable inhibitor of uncoupling protein 2 (UCP2) [22]. After adding genipin to chondrocytes, UCP2 expression decreased. This effect was increased with both concentration and time duration of genipin (Figure 5A). In the 100 um/ml 48 hour and 200 um/ml 24 hour groups, ROS production did not change obviously after adding genipin; in the 200 um/ml 48 hour group, ROS production increased (Figure 5B). That same group was the only one to display loss of chondrocyte phenotype when compared to the control group. The expression of col2, Sox9 and aggrecan decreased, and col1 expression increased in the 200um/ml 48 hour group (Figure 5C). The expression of col1 protein was also increased, and the expression of col2 protein was decreased in the 200um/ml 48 hour group (Figure 5D). The SD rats displayed similar results after genipin injections to the knee: col1 expression increased, col2 and aggrecan expression decreased, and the chondrocyte phenotype had been lost according to our immunohistochemical technique (Figure 5E, 5F). These results indicate that UCP2 can maintain chondrocyte phenotype by suppressing ROS production.

### Inhibiting NOX1/4 expression can relieve the loss of chondrocyte phenotype and decrease ROS production

**Figure 6:**
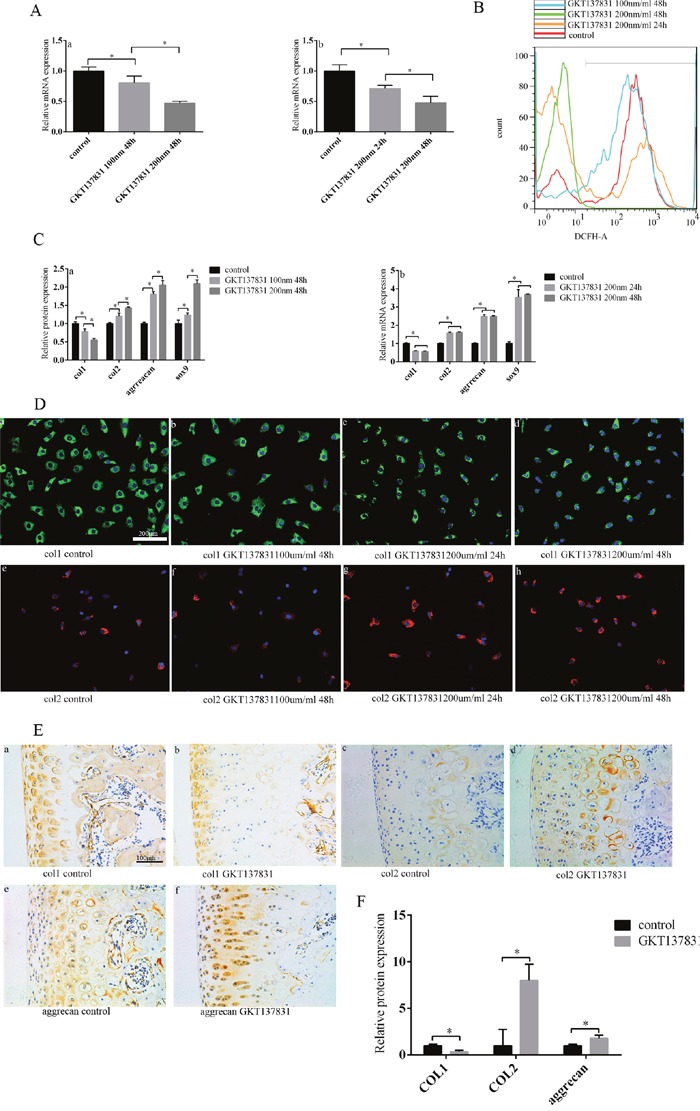
Inhibiting NOX1/4 expression can decrease ROS production, and thus inhibit the loss of chondrocyte phenotype **(A)** After increasing GKT137831 concentration and duration, NOX1/4 expression decreased. **(a)** The relationship between GKT137831 concentration and NOX1/4 expression. **(b)** The relationship between GKT137831 duration and NOX1/4 expression. **(B)** After increasing GKT137831 concentration and duration, ROS production decreased as determined by cell flow technology. **(C)** After increasing GKT137831 concentration and duration, chondrocyte phenotype loss was inhibited according to PCR. **(a)** The relationship between GKT137831 concentration and chondrocyte phenotype. **(b)** The relationship between GKT137831 duration and chondrocyte phenotype. **(D)** After inhibiting NOX1/4 expression, col1 expression decreased and col2 expression increased according to cell fluorescence. **(a/b/d)** The relationship between col1 expression and GKT137831 concentration. **(a/c/d)** The relationship between col1 expression and GKT137831 duration. **(e/f/h)** The relationship between col2 expression and GKT137831 concentration. **(e/g/h)** The relationship between col2 expression and GKT137831 duration. Bar = 200 μm. **(E)** The loss of chondrocyte phenotype was inhibited after injecting GKT137831 into the knee joint of SD rats, as determined by immunohistochemistry. **(a/b)** Col1 expression. **(c/d)** Col2 expression. **(e/f)** Aggrecan expression. Bar = 100 μm. **(F)** Immunohistochemistry results are the mean ± SD, and are representative of three independent experiments.

GKT137831 is a novel and specific dual Nox1/Nox4 inhibitor [23, 24]. Chondrocytes cultured *in vitro* were divided into several groups and received different doses of NOX1/4 inhibitor GKT137831 for different amounts of time. After adding GKT137831, NOX1/4 expression decreased as concentration and duration increased, according to PCR (Figure 6A). ROS production decreased in all four groups after adding GKT137831 (Figure 6B). Compared with the control group, loss of chondrocyte phenotype was inhibited (col1expression was decreased, col2, Sox9, and aggrecan increased) after adding GKT137831. Chondrocyte phenotype loss was inhibited in both the 100 nm/ml and 200 nm/ml 48 hour groups, more so in the higher dose group (Figure 6C). According to PCR, col1 expression was decreased, col2, Sox9, and aggrecan were increased after 24 and 48 hours of 200 nm/ml GKT137831 compared with the control group, with no difference between the two time groups (Figure 6C). Protein levels differed from gene expression observed: col1 expression decreased and col2 increased with increased GKT137831 concentration and duration (Figure 6D). In our animal model, loss of chondrocyte phenotype was suppressed in the SD rats injected with GKT137831 compared with the control group according to immunochemistry (Figure 6E, 6F). These results confirm that inhibiting NOX1/4 expression can maintain chondrocyte phenotype by decreasing ROS production.

### Inhibiting NOX1/4 expression can increase UCP2 expression

**Figure 7:**
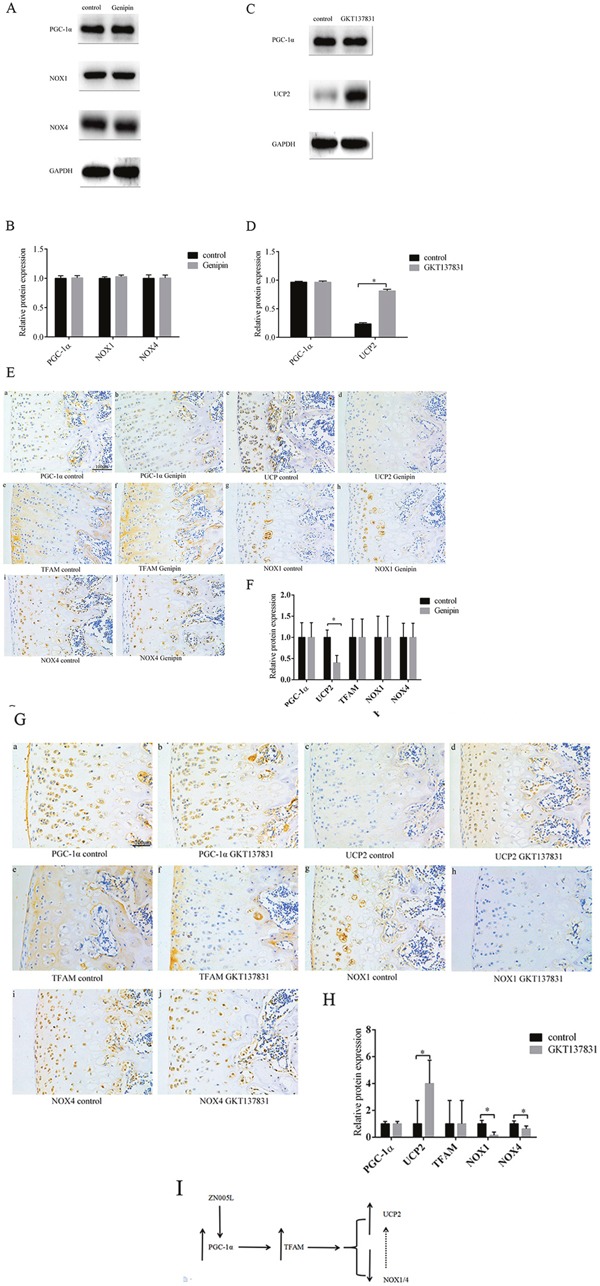
Inhibiting NOX1/4 expression can increase UCP2 expression **(A/B)** The expression of PGC-1α and NOX1/4 was not changed after UCP2 expression was inhibited, according to Western blot. Values are the mean ± SD, and are representative of three independent experiments. **(C/D)** UCP2 expression increased and PGC-1α did not change after inhibiting NOX1/4 expression, according to Western blot. Values are the mean ± SD, and are representative of three independent experiments. **(E/F)** The expression of PGC-1α, TFAM, and NOX1/4 did not change after injecting genipin into the knee joint cavity of SD rats, according to immunohistochemistry. **(a/b)** PGC-1α expression. **(c/d)** UCP2 expression. **(e/f)** TFAM expression. **(g/h)** NOX1 expression. **(i/j)** NOX4 expression. Bar = 100 μm. Values are the mean ± SD of three independent experiments. **(G/H)** The expression of PGC-1α and TFAM did not change and UCP2 increased after injecting GKT137831 into the knee joint cavity of SD rats according to immunohistochemistry. **(a/b)** PGC-1α expression. **(c/d)** UCP2 expression. **(e/f)** TFAM expression. **(g/h)** NOX1 expression. **(i/j)** NOX4 expression. Bar = 100 μm. Values are the mean ± SD of three independent experiments. **(I)** Pathway diagram.

After adding UCP2 inhibitor genipin, the expression of PGC-1α and NOX1/4 did not change according to Western blot (Figure 7A, 7B). Next, after adding inhibitor GKT137831 into the medium, the PGC-1α and TFAM expression did not change, but UCP2 increased (Figure 7C, 7D). If UCP2 is suppressed, no change occurs to the expression of PGC-1α, TFAM, and NOX1/4 (Figure 7E, 7F). UCP2 was higher in the knee cartilage of SD rats injected with GKT137831, but PGC-1α and TFAM expression did not change (Figure 7G, 7H). This suggests inhibiting NOX1/4 expression can increase UCP2 expression and further inhibit the loss of chondrocyte phenotype. Lastly, UCP2 and NOX1/4 did not produce negative feedback as the downstream factors of PGC-1α-TFAM pathway (Figure 7I)

## DISCUSSION

Chondrocytes of OA patients show higher ROS levels, resulting in more severe changes in chondrocyte phenotype (cartilage matrix structure and function) [25, 26]. Alternatively, a reduction in oxygen tension in monolayer cultures induces the re-differentiation of dedifferentiated chondrocytes, inhibiting the loss of chondrocyte phenotype [10], consistent with the idea that low oxygen tension protects cells from the onset of senescence [27, 28]. We suggest that the loss of chondrocyte phenotype is accompanied by increased ROS production (Figure 1A, 1B), consistent with previous studies. Our experimental results further indicate that increased NOX1/4 and decreased UCP2 in chondrocytes can promote the loss of chondrocyte phenotype by increasing ROS production.

PGC-1α can increase ATP production and reduce ROS production by altering the structure of mitochondrial respiratory complexes [19, 29, 30]. In our experiments, stimulated PGC-1α expression could inhibit the loss of chondrocyte phenotype (Figure 3C). This indicates that PGC-1α can maintain the chondrocyte phenotype. In the ZN005L+siTFAM group, NOX1/4 expression did not change, and UCP2 was marginally increased when compared with the ZN005L group (Figure 4E). The PGC-1α-TFAM pathway can reduce ROS production and subsequently reverse the loss of chondrocyte phenotype by decreasing and increasing NOX1/4 and UCP2 expression, respectively.

Unlike NOX1/4, UCP2 expression was increased slightly in the ZN005L+ siTFAM group when compared with the ZN005L group (Figure 4E). It may be that PGC-1α can regulate UCP2 expression outside of the TFAM pathway. Indicators of chondrocyte phenotype (col2 and aggrecan) were increased and ROS production was decreased in the ZN005L+siTFAM group when compared with the siTFAM group (Figure 4B, 4C). For the siTFAM+ZN005L group, activating PGC-1α can reduce ROS production by increasing the expression of UCP2, which increases col2 and aggrecan expression. However, unlike col2 and aggrecan, col1 and sox9 were not changed (Figure 4C). Perhaps col2 and aggrecan are more sensitive to changes of ROS, while col1 and Sox9 expression is more stable. We suspect that col1 and sox9 are affected by ATP levels, while col2 and aggrecan expression are affected by ROS production.

We further explored the relationship between UCP2 and NOX1/4 in the cartilage tissue. The expression of UCP2 was increased after inhibiting NOX1/4 expression in chondrocytes, suggesting that the reduction of NOX1/4 reduced ROS production (Figure 7C). However, inhibiting UCP2 expression did not affect NOX1/4 expression, suggesting the lack of a feedback relationship (Figure 7A).

The loss of chondrocyte phenotype results in decreased cartilage function, which leads to osteoarthritis. The incidence of osteoarthritis increases every year in China [31, 32], so maintaining chondrocyte function is important to more and more patients. Our previous study found that inhibiting the Sirt1 pathway accelerated cartilage senescence in knockout (KO) mice [33]. PGC-1α is downstream of Sirt1, and PGC-1α can regulate ROS production. Here we conducted a more in-depth study, and showed activating the PGC-1α-TFAM pathway can reduce ROS production and reverse the loss of chondrocyte phenotype by increasing and decreasing UCP2 and NOX1/4 expression, respectively.

## MATERIALS AND METHODS

### Chondrocyte isolation

Fresh joints were obtained from Sprague–Dawley (SD) rats (4 weeks old). Articular cartilage was harvested and subjected to trypsin/collagenase digestion to isolate chondrocytes, as previously described [6]. Articular chondrocytes were cultured *in vitro* by adding DMEM/F12 supplemented with 10% FBS (Gibco; lot number 1652790), penicillin/streptomycin (50,000 U/50 mg), and L-glutamine (4.5 mM) and passaged when the cartilage cells rise to 80% confluency.

### Flow cytometry

One million chondrocytes (of each processing group) were washed in PBS and incubated for 0.5 h at 37°C with DCFH-DA-FITC. Cells were centrifuged at 200xg, the supernatants were removed, and the cells were washed three times in PBS. The labelled cells were resuspended in 1 ml of PBS, and analyzed by flow cytometry.

### Reverse transcription and quantitative real-time PCR

RNA was extracted from chondrocytes using TRIzol Reagent (Invitrogen), and was subject to cDNA synthesis using the qScript cDNA synthesis kit (Quanta Biosciences, Gaithersburg, MD, USA) following the manufacturer's instructions. Real time quantitative PCR was performed using PerfeCTa SYBR Green FastMix (Quanta Biosciences). Standard recommended PCR protocols were performed (50°C for 2 min, 94°C for 10 min, 95°C for 30 sec, 60°C for 1 min, with steps 3 and 4 repeated for 40 cycles) using the ABI 7900 HT Fast Real-Time PCR System (Applied Biosystems, Carlsbad, CA, USA). Oligonucleotides are shown in Supplementary Table 1.

### Immunocytochemistry

To immunostain cells, the samples were fixed in 4% paraformaldehyde in PBS for 10 min at room temperature. After the cells were washed three times in PBS/0.1% BSA for 5 min, they were permeabilized using 0.2% Triton (Sigma; T9284) in PBS for 20 min, and then washed in PBS/0.1% BSA. Primary antibodies against Col2 and Col1 (Abcam, UK) were diluted 1/150 in PBS/0.1% BSA and incubated overnight at 4°C. After the samples were washed, the cells were incubated with a FITC-conjugated goat anti-rabbit secondary antibody (1:500; Abcam) and DAPI (Sigma) for 1 h at room temperature. Fluorescent images were obtained using a Nikon A1-R inverted confocal microscope.

### Animal model

Left knee joints of SD rats were injected with 100 um/ml ZN005L (PGC-1 agonist), or Genipin 100 um/ml (UCP2 inhibitor), or GKT137831 100 nm/ml (Nox1/4 inhibitor), and the right knee joints were used as controls. Animals were injected weekly for four weeks, and then the knee joint cartilage of both knees were tested by immunohistochemistry. In addition, the articular cartilage of 24-week rats was tested by immunohistochemistry.

### Immunohistochemistry

Knee cartilage was de-paraffinized and rehydrated and then subjected to antigen retrieval by incubating the tissues in hot (95°C) sodium citrate buffer (0.01 M, pH 6.0) for 10 min. The tissue sections were exposed to hydrogen peroxide (3% H_2_O_2_) for 5 min to quench the endogenous peroxidase, and then blocked in 30% horse serum for 30 min. The slides were incubated overnight at 4°C with col2, col1, aggrecan, PGC-1α, TFAM, UCP2, NOX1, and NOX4 antibodies (1:150 dilution; Abcam, UK). Non-immune mouse IgG was used as a negative control. After the tissues were washed with 1X tris-buffered saline containing 0.1% Tween-20 (TBST), the slides were incubated with biotinylated secondary antibodies (anti-goat IgG; Santa Cruz) and analyzed.

### Chondrocyte inhibitors and agonists

Media was removed from chondrocytes at approximately 70% confluency and replaced with media containing ZN005L(100 um/ml or 200 um/ml), or Genipin (100 um/ml or 200 um/ml), or GKT137831(100 nm/ml or 200 nm/ml), or siTFAM + 100 um/ml ZN005L. 24h and 48h were time points of interest in each group.

### siRNA-mediated gene knockdown

Cells were seeded on 6 well plates in advance of 24h replacement of fresh cell culture solution, and 55%∼65% density of the treated cells were transfected. Next, 125 ul no serum sugar DMEM was added, then 7.5 ul of TFAM siRNA or negative-control siRNA, incubated for 5 minutes at room temperature. In another set of wells, 125 ul no serum sugar DMEM was added, followed by 7 ul of transfection agent, incubated for 5 minutes at room temperature. The siRNA solution was mixed with the transfection solution at room temperature for 20 minutes to form the siRNA-liposome complex. The cells were rinsed with warm PBS two to three times, then 250 ul of the compound solution was added to the appropriate wells. After incubating at 37°C for 6 h, the medium was replaced with normal cell culture medium, then incubated for 48 – 72 h. Experimental group siRNA and negative control group siRNA were purchased from GenePharma, China, and transfection reagent ExcelDeliver was purchased from EarthOx, USA.

### Western blot analysis

Cells were sonicated in standard lysis buffer containing protease and phosphatase inhibitors. Western blots were performed using standard protocols, and proteins were visualized using Pierce West Dura detection reagent and a Chemi Doc-It Imaging System attached to a Biochemi HR camera. To measure protein abundance, the scanned images were quantitated using ImageJ Plus software (National Institutes of Health, Bethesda, MD, USA). Before comparisons were made, each target protein was normalized to the value for GAPDH.

### Statistical analysis

All *in vitro* experiments were repeated at least three times, and different samples were used for each experimental replicate. The results from the *in vitro* experiments were analyzed using one-way analysis of variance (ANOVA) or t-tests if only two conditions were being compared. All data were analyzed using Prism V.5.0b software (GraphPad Software, USA). P-values ≤ 0.05 were considered statistically significant. The results are expressed as the means ± S.D.

## SUPPLEMENTARY MATERIALS TABLE


