# Supplementary material for: Increasing UCP2 expression and decreasing NOX1/4 expression maintain chondrocyte phenotype by reducing reactive oxygen species production
Source: Oncotarget. 2017 Jul 1;8(38):63750–63. doi: 10.18632/oncotarget.18908 (PMC5609958; doi:10.18632/oncotarget.18908)
Supplement: Supplementary file 1 [file oncotarget-08-63750-s001.pdf]

Increasing UCP2 expression and decreasing NOX1/4 expression maintain chondrocyte phenotype by reducing reactive oxygen species production

SUPPLEMENTARY MATERIAL

Supplementary Table 1: Oligonucleotides for real-time quantitative PCR analysis

Gene	Sense (5'-3')	Antisense (5'-3')
Col1	cgagggccaagacgaagacatc	gggcagacgggacagcactc
Col2	ctcacgccttcccattgtt	atcaggtcaggtcagccattc
Aggrecan	cctgctacttcatcgacccc	agatgctgttgactcgaacct
Sox9	cccttcgtggaggaggcgga	ccggaggaggagtgtggcga
PGC-1α	tacagacaccacacacatcg	cctttcgtgctcattggctt
TFAM	cgcctgtcacgccttatctgta	tgcatctgggtgtttagctta
UCP2	acaagaccattgcacgagag	catggtcagggcacagtggc
NOX1	tcactaacgtgtgggtcagc	gctctcatgttgccaaagcc
NOX4	gaacccaagttccaagctca	gcacaaaggtccagaaatcc
GAPDH	tcaccaccatggagaaggc	gctaagcagttggtggtgca
